# Screen Time and Parent-Child Talk When Children Are Aged 12 to 36 Months

**DOI:** 10.1001/jamapediatrics.2023.6790

**Published:** 2024-03-04

**Authors:** Mary E. Brushe, Dandara G. Haag, Edward C. Melhuish, Sheena Reilly, Tess Gregory

**Affiliations:** 1 Telethon Kids Institute, University of Western Australia, Adelaide, South Australia, Australia; 2 School of Public Health, University of Adelaide, Adelaide, South Australia, Australia; 3 Department of Education, University of Oxford, Oxford, United Kingdom; 4 Menzies Health Institute Queensland, Griffith University, Gold Coast, Queensland, Australia

## Abstract

**Importance:**

Growing up in a language-rich home environment is important for children’s language development in the early years. The concept of “technoference” (technology-based interference) suggests that screen time may be interfering with opportunities for talk and interactions between parent and child; however, limited longitudinal evidence exists exploring this association.

**Objective:**

To investigate the longitudinal association between screen time and 3 measures of parent-child talk (adult words, child vocalizations, and conversational turns) when children are 12 to 36 months of age.

**Design, Setting, and Participants:**

This Australian prospective cohort study used advanced speech recognition technology to capture young children’s screen time and home language environment, on an average 16-hour day. Data were collected from 220 families once every 6 months in the family home when children were 12, 18, 24, 30, and 36 months of age, from January 1, 2018, to December 31, 2021. Statistical analysis took place from November 1, 2022, to July 31, 2023.

**Exposure:**

Language Environment Analysis (LENA) technology provided automated counts of children’s language environment and exposure to electronic noise. The exposure of interest was screen time, which was calculated based on manual coding of LENA electronic noise audio segments.

**Main Outcomes and Measures:**

Three measures of parent-child talk were outcomes of focus: adult words, child vocalizations, and conversational turns. Separate models were run for each of the 3 outcomes and included adjustment for child sex, child age, maternal educational level, number of children at home, number of home activities, and primary caregiver’s psychological distress.

**Results:**

The study included 220 families (120 girls [54.6%]; mean [SD] gestational age of children, 39.3 [1.5] weeks; mean [SD] age of mother at childbirth, 31.3 [4.8] years). Adjusted linear mixed-effect models demonstrated that increases in screen time were associated with decreases in measures of parent-child talk. The largest decreases were seen at 36 months, when an additional minute of screen time was associated with a reduction of 6.6 (95% CI, −11.7 to −1.5) adult words, 4.9 (95% CI, −6.1 to −3.7) child vocalizations, and 1.1 (95% CI, −1.4 to −0.8) conversational turns.

**Conclusion and Relevance:**

Findings of this study support the notion of technoference for Australian families, whereby young children’s exposure to screen time is interfering with opportunities to talk and interact in their home environment. This finding has implications for interventions and supports aimed at promoting a language-rich home environment, with families needing support in understanding the potential association of screen time with opportunities for children and adults to talk and interact in their home environment.

## Key Points

**Question:**

What is the association between screen time and adult words spoken, child vocalizations, and conversational turns when children are 12 to 36 months of age?

**Findings:**

This cohort study found a negative association between screen time and measures of parent-child talk across those early years. For every additional minute of screen time, children heard fewer adult words, spoke fewer vocalizations, and engaged in fewer back-and-forth interactions.

**Meaning:**

This study suggests that screen time is a mechanism that may be getting in the way of children experiencing a language-rich home environment during the early years; interventions aiming to promote early use of language should include support to manage screen time.

## Teaser

This cohort study investigates the longitudinal association between screen time and 3 measures of parent-child talk (adult words, child vocalizations, and conversational turns) when children are 12 to 36 months of age.

## Introduction

The importance of a language-rich home environment during the early years of life has been well established.^1^ Existing evidence indicates positive associations between early language exposure and children’s language development,^2,3,4,5^ socioemotional development,^6^ IQ,^7^ and brain function.^8,9,10^ As a result, programs aiming to increase the amount of parent-child talk in young children’s homes have become increasingly popular.^11,12,13^ Estimates of the home language environment in Australia and the US have shown large variability among families.^14,15,16^ Talking with children may seem an easy and simple activity; however, in the busy lives of families, it may be anything but simple. It is crucial to investigate potential factors within the home environment that may interrupt parents’ opportunities to talk and interact with their children to help inform interventions aimed at building a language-rich home environment and, in turn, support children’s language development.

A growing body of evidence has examined the associations between screen time and parent-child talk, which encompasses adult words, child vocalizations, and back-and-forth interactions.^17,18^ The phenomenon coined “technoference” (technology-based interference) suggests that parents’ time using screen-based devices interferes with daily opportunities to talk and respond to their child.^19^ A recent systematic review demonstrated that parental smartphone use was negatively associated with parental responsiveness and attention toward their children aged 3 years or younger.^17^ Another systematic review investigating parental use of mobile computing devices and the social and emotional development of children aged 10 years or younger found less engagement, harsher responses, and fewer verbal and nonverbal communications between parents and children when parents were using a mobile device.^20^ Nonetheless, many of the studies cited in the systematic reviews have considered screen time only within limited context (eg, during meals, at an outdoor playground) and are often cross-sectional. They have also focused on parent’s screen time (usually mobile telephone use), rather than considering both the adult’s and child’s screen time across a range of devices. Finally, many studies rely heavily on parent-reported measures of screen time and interaction or responsiveness, which may be less accurate and prone to socially desirable responses compared with objective measures.

One exception is a study that used speech recognition technology to understand the association of audible television with adult words, child vocalizations, and conversational turns among children aged 2 to 48 months (n = 329).^18^ This study compared the number of vocalizations and conversational turns that an individual child experienced on the number of days with high exposure to television with the number of days with low exposure to television and found that audible television was associated with reductions in age-adjusted *z* scores for child vocalizations (−0.3 [95% CI, −0.3 to −0.2]) and conversational turns (−0.2 [95% CI, −0.3 to −0.2]). It also demonstrated that for every additional hour of audible television, adults spoke 770 fewer words (95% CI, −1004 to −535 words) to their child. That study was published in 2009, prior to the increase in mobile technology use, which has likely changed the way that screen time is associated with parent-child talk compared with television exposure only.

The present study aims to understand the longitudinal association between a child’s screen exposure and 3 measures of parent-child talk: (1) adult words, (2) child vocalizations, and (3) parent-child interactions (or conversational turns), in the first 3 years of life. This research builds on existing literature by using data from a recent prospective cohort study that began in 2017 and uses a novel approach to measuring screen time.^14,15^

## Methods

### Study Design

The Language in Little Ones (LiLO) study is a prospective cohort study (n = 302) that collected data biannually from 6 months of age until children reached school age (approximately 5 years of age). The LiLO study aimed to understand young Australian children’s home language environment, quantified by the amount of language children heard and spoke. Parent-child talk was captured once every 6 months, for 16 hours, using advanced speech recognition technology called Language Environment Analysis (LENA). The LENA technology automatically quantified the number of adult words, the number of vocalizations made by the child, and the number of conversational turns between the adult and child. LENA also calculated the amount of time children were exposed to television or electronic noise. Following stakeholder and community interest in better understanding screen time in early childhood, a nested study of LiLO was established called the Electronic Use in Little Ones (EUiLO) study. The EUiLO study focused on coding the television or electronic noise data that had already been collected in the first 3 years of the LiLO study, from January 1, 2018, to December 31, 2021, to provide a measure of screen exposure, which differentiated between screen time and other electronic sounds (ie, electronic appliances). Participants were compensated with a $10 supermarket voucher after each wave of data collection. The University of Western Australia human research ethics committee granted ethics approval for both studies, and participants provided informed written consent. The Strengthening the Reporting of Observational Studies in Epidemiology (STROBE) reporting guideline for cohort studies was followed in the preparation of this article.^21^

### Participants

Families were recruited antenatally and postnatally for the LiLO study across South Australia, Western Australia, and Queensland. Further details on recruitment efforts have been previously reported.^14^ Eligibility criteria included (1) children born in 2017, (2) predominately English spoken in the home due to the validity of the LENA technology at the time of recruitment, (3) children born at 37 weeks’ or more gestation, (4) singleton children, and (5) the child did not have a diagnosed cause of language impairment. A key focus of the LiLO study was to understand socioeconomic inequalities in early language, and as such there was an additional eligibility criterion focused on maternal educational level. Mothers who completed a university degree (bachelor’s degree or higher) were classified into a high education group, and mothers who had no postsecondary school education were classified into a low education group. Mothers whose educational level could not be categorized into either group (ie, those with certificate-level qualifications) were ineligible for the study.

A total of 302 families participated in the LiLO study. Retrospective consent was sought from participants still active in the LiLO study in 2020 (n = 277) to undertake the additional coding by researchers (see Procedures subsection) and the analyses of television or electronic noise data for the EUiLO study. A total of 55 families did not consent to the EUiLO study, and 2 families were also removed from the study due to their child developing a diagnosed cause of language impairment, which left a total of 220 families in the analysis sample for the present study (Figure).

**Figure.**
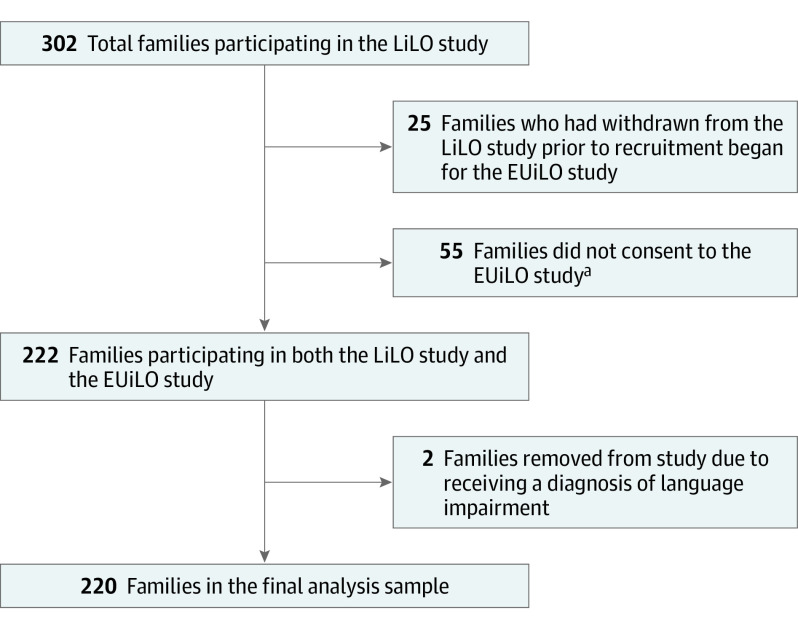
Flowchart of Participation for the Final Analysis Sample EUiLO indicates Electronic Use in Little Ones; and LiLO, Language in Little Ones. ^a^Not consenting to participate in the EUiLO study meant that these participants did not have valid data on screen time.

### Measures

The exposure (screen time) and outcome measures (3 measures of parent-child talk) were captured using the LENA technology. This technology includes a specially designed t-shirt or vest with a pocket in the front to hold a small digital language processor (DLP), which records all the audio around the child for 16 hours. The LENA software then processes the audio through algorithmic speech signal analysis and provides automated counts of adult words, child vocalizations, conversational turns, and exposure to television or electronic noise.^22^ The automated counts for adult words include any words spoken within an approximately 3-m radius of the child wearing the LENA DLP, whether directed at the child or not. The families were instructed to choose an average home day for the child to wear the t-shirt and DLP. This could not be a day when the child attended childcare, when the child was sick, or when the child was going to attend loud public events (eg, a sporting match). Evidence suggests good reliability of the LENA technology, with high consistency between counts generated by LENA and human transcribers for each measure of parent-child talk.^23^ The level of agreement, however, was lower for television or electronic noise data (71%) compared with language measures (eg, 81% for adult words).

Child and family characteristics that may be associated with the link between screen time and parent-child talk were determined a priori and measured via parent report at the biannual home visit. The confounders included in the study were child’s sex (male or female), child’s age in months (to account for any variation in the exact age the child completed the LENA recording day), mother’s highest level of education (high or low), primary caregiver’s self-reported psychological distress (measured using the Kessler 6-question scale,^24^ where a score of 0-7 indicates low distress; a score of 8-12, moderate distress; and a score of 13-24, high distress), the mean number of home activities (eg, singing, outdoor play) completed with the child, and the number of other children living in the home.

### Procedures

Data from the second to the sixth wave of data collection in the LiLO study and the EUiLO study were collected between January 1, 2018, and December 31, 2021. Once every 6 months, a researcher would visit the family home and complete the standardized questionnaires and demonstrate the use of the LENA equipment. The family was then asked to complete their “recording day” within approximately 2 weeks, before the researcher returned to collect the equipment. The audio recording was uploaded to the LENA software, automatically providing counts of parent-child talk variables and television or electronic noise exposure. As part of participation in the EUiLO study, researchers would export the audio in 5-minute segments when LENA flagged electronic noise during the 16-hour day. Researchers then listened to these audio segments to assess the type of electronic noise (eg, was the child exposed to a screen or a microwave beeping?). Within each segment, both parent-child talk and electronic noise could be occurring simultaneously. If language was occurring, this was captured separately in the automatic counts of parent-child talk variables. Therefore, the coding of each segment focused on categorizing the electronic noise as screen media, music, noise, or sleeping based on audio cues, such as media content theme songs or character voices, identification of a familiar noise (eg, car starting or microwave), contextual conversations (eg, the child asks for iPad), and what was reported in an accompanying activity diary completed by the parent. When the source of the noise could not be determined by multiple coders, it was classified as unknown. If, within the 5-minute segment, the child was not exposed to screen media for the full duration, the exact number of minutes and seconds (rounding to the closest 5 seconds) would be recorded. All research staff were trained by a master coder and were required to achieve 90% accuracy before coding independently. Screen media were used as the primary measure of screen time and included the time the child was exposed to any screen-based device, excluding when the child was asleep. The recording day procedure and coding method were consistently applied across all waves of data collection.

### Statistical Analysis

Statistical analysis took place from November 1, 2022, to July 31, 2023. Analyses for the present study focused on data collected over 5 waves of the study, when the children were 12, 18, 24, 30, and 36 months of age. These ages were the focus because the exposure, outcome, and all confounders were available for these waves of data collection. To estimate the association between children’s screen exposure and parent-child talk variables at each wave of data collection, linear mixed-effect models were used to account for both the within-person and between-person variability in the repeated measures data, using the mixed command in Stata, version 17 (StataCorp).^25^ Separate unadjusted and adjusted models were run for each of the parent-child talk outcomes of interest: (1) adult words, (2) child vocalizations, and (3) conversational turns. An interaction between the amount of screen exposure and the wave of data collection was included as a fixed effect, to understand differences across waves. Participant identification was included as a random effect, to account for the variation among participants. In each of the adjusted models, child sex, child’s age in months, mother’s highest level of education, primary caregiver’s self-reported psychological distress, the mean number of home activities, and the number of other children in the home were controlled for. The parameters were computed using the restricted maximum likelihood function, to account for missing data across the study, and the residuals were modeled under the unstructured variance-covariance structure, to account for distinct variances and covariances among the random effects.

## Results

The study included 220 families (120 girls [54.6%]; mean [SD] gestational age of children, 39.3 [1.5] weeks; mean [SD] age of mother at childbirth, 31.3 [4.8] years) (Table 1). Approximately half the sample included the first-born child (109 [49.5%]). A total of 133 mothers (60.5%) were categorized into the high education group, and 190 mothers (86.4%) were working until their pregnancy.

**Table 1.** Sociodemographic Characteristics of the Sample

Characteristic	No. (%) (N = 220)
Child	
Girls	120 (54.6)
Gestation, mean (SD), wk	39.3 (1.5)
Firstborn	109 (49.5)
Mother	
Highest level of completed education is university	133 (60.5)
Age at childbirth, mean (SD), y	31.3 (4.8)
Working until pregnancy	190 (86.4)
No. of home activities with child at 12 mo, mean (SD)^a^	2.1 (0.4)
Psychological distress score at 12 mo, mean (SD)^b^	3.8 (2.9)

^a^ A 4-point Likert scale was used in the Longitudinal Study of Australian Children (0 = none; 1 = 1-2 days; 2 = 3-5 days; 3 = every day or 6-7 days).

^b^ Measured using the Kessler 6-question scale (0-7, low distress; 8-12, moderate distress; 13-24, high distress).

Table 2 shows the distribution of screen time and each parent-child talk measure at each age. When children were 12 months of age, they were exposed to a mean (SD) 87.8 (107.6) minutes (ie, 1 hour, 28 minutes) of screen time, heard a mean (SD) of 14 997.8 (6873.4) adult words, produced a mean (SD) of 1394.7 (522.7) vocalizations, and engaged in a mean (SD) of 369.4 (167.4) conversational turns per day. Screen time, child vocalization, and conversational turn counts increased as children got older, whereas the number of adult words remained relatively stable across time, only increasing slightly as children grew up. By 36 months of age, children were exposed to a mean (SD) of 172.1 (134.7) minutes (ie, 2 hours, 52 minutes) of screen time, heard a mean (SD) of 16 302.6 (6654.7) adult words, produced a mean (SD) of 3306.7 (1612.8) vocalizations, and engaged in a mean (SD) of 734.4 (404.2) conversational turns per day.

**Table 2.** Descriptive Statistics for Screen Time and Parent-Child Talk Variables at Each Time Point for 220 Families^a^

Time point	Mean (SD) value			
	Screen time, min	Adult words, No.	Child vocalizations, No.	Conversational turns, No.
12 mo	87.8 (107.6)	14 997.8 (6873.4)	1394.7 (522.7)	369.4 (167.4)
18 mo	118.0 (111.2)	14 987.4 (6884.2)	2976.4 (803.8)	541.3 (289.1)
24 mo	147.2 (123.4)	15 980.5 (6469.5)	2771.3 (1214.3)	709.3 (384.9)
30 mo	165.9 (123.1)	15 793.6 (6423.5)	3405.9 (1558.9)	750.2 (385.7)
36 mo	172.1 (134.7)	16 302.6 (6654.7)	3306.7 (1612.8)	734.4 (404.2)

^a^ Screen time is reported in number of minutes across the Language Environment Analysis (LENA) recording day. Adult words, child vocalizations, and conversation turns are reported as number of words, vocalizations, or turns across the LENA recording day.

Results of the unadjusted mixed-effect models indicated an overall negative association between the amount of screen time children were exposed to and the number of adult words children heard at all ages (Table 3). For instance, at 18 months, each additional minute of screen time was associated with children hearing 12.0 (95% CI, −17.4 to −6.5) fewer adult words. For child vocalizations, the association was less clear and appeared to change over time, with an additional minute of screen time associated with a decrease of 1.9 (95% CI, −2.7 to −1.2) vocalizations at 12 months and an increase of 2.2 (95% CI, 1.1-3.4) vocalizations at 30 months. For conversational turns, there was a negative association with screen time, with 1 additional minute of screen time associated with a decrease of 0.6 (95% CI, −0.9 to −0.4) conversational turns at 12 months and a decrease of 0.3 (95% CI, −0.6 to −0.1) conversational turns at 18 months. As the children aged, however, these decreases disappeared.

**Table 3.** Unadjusted and Adjusted Linear Mixed-Effect Models for the Association Between Parent-Child Talk Variables and the Amount of Screen Time From 12 to 36 Months (N = 220)

Measure	Unadjusted model		Adjusted model^a^	
	β (95% CI)	*P* value	β (95% CI)	*P* value
**Adult words**				
Intercept, No.	16 476.8 (15 664.6 to 17 288.9)	<.001	10 165.4 (7057.7 to 13 273.2)	<.001
Screen time at child age				
12 mo	−5.2 (−12.5 to 2.2)	.17	5.3 (−2.8 to 13.5)	.20
18 mo	−12.0 (−17.4 to −6.5)	<.001	−6.7 (−12.4 to −0.9)	.02
24 mo	−5.3 (−9.6 to −0.9)	.02	−3.1 (−7.6 to 1.3)	.17
30 mo	−5.9 (−10.1 to −1.8)	.01	−6.1 (−10.5 to −1.6)	.01
36 mo	−4.7 (−8.9 to −0.6)	.03	−6.6 (−11.7 to −1.5)	.01
**Child vocalizations**				
Intercept, No.	1777.8 (1686.9 to 1868.8)	<.001	−240.4 (−644.9 to 164.1)	.24
Screen time at child age				
12 mo	−1.9 (−2.7 to −1.2)	<.001	−0.4 (−1.1 to 0.4)	.32
18 mo	−0.6 (−1.3 to 0.2)	.13	−1.3 (−2.0 to −0.5)	<.001
24 mo	0.6 (−0.4 to 1.6)	.22	−1.9 (−2.8 to −0.9)	<.001
30 mo	2.2 (1.1 to 3.4)	<.001	−1.9 (−3.1 to −0.8)	<.001
36 mo	1.5 (0.4 to 2.6)	.01	−4.9 (−6.1 to −3.7)	<.001
**Conversational turns**				
Intercept, No.	470.7 (441.8 to 449.6)	<.001	52.5 (−68.2 to 173.3)	.39
Screen time at child age				
12 mo	−0.6 (−0.9 to −0.4)	<.001	−0.2 (−0.4 to 0.03)	.10
18 mo	−0.3 (−0.6 to −0.1)	<.001	−0.3 (−0.6 to −0.1)	.01
24 mo	−0.02 (−0.3 to 0.3)	.89	−0.4 (−0.6 to −0.1)	.01
30 mo	0.2 (−0.4 to 0.5)	.10	−0.6 (−0.9 to −0.3)	<.001
36 mo	0.2 (−0.1 to 0.4)	.25	−1.1 (−1.4 to −0.8)	<.001

^a^ Adjusted models include child sex, child age (in months), maternal educational level (high or low), number of children at home, mean number of home activities, and primary caregiver’s psychological distress.

In the adjusted models, the results show that increases in screen time were associated with decreases in parent-child talk across all variables and ages, with the only exception being screen time at 12 months having no association with the 3 parent-child talk outcomes (Table 3). The largest associations were seen at 36 months, when an additional minute of screen time was associated with a reduction of 6.6 (95% CI, −11.7 to −1.5) adult words, 4.9 (95% CI, −6.1 to −3.7) child vocalizations, and 1.1 (95% CI, −1.4 to −0.8) conversational turns.

## Discussion

The present study used data from a prospective cohort study that analyzed longitudinal data on children between 12 and 36 months of age to examine the association between child screen time and 3 measures of parent-child talk (adult words, child vocalizations, and conversational turns). Findings from the mixed-effects models indicated that for every additional minute of screen exposure, parents and children were generally talking or vocalizing less and were engaging in fewer back-and-forth interactions. This association was less clear in the unadjusted models, with no associations evident at some time points in the child vocalizations and conversational turn outcome models. In the adjusted models, which took into account several child and family confounders, a negative association between screen time and parent-child talk became clear, highlighting the important role that maternal educational level, child sex, primary caregiver’s psychological distress, and number of home activities play. Specifically, at 36 months of age in the adjusted models, for 1 extra minute of screen time, children heard 6.6 fewer adult words, made 4.9 fewer vocalizations, and engaged in 1.1 fewer conversational turns. This finding aligns with the concept of technoference and the existing literature, which has suggested that increases in screen time decrease parent-child interactions.^17,18,20^

For families who follow the current World Health Organization screen time guidelines (eg, 1 hour per day at 36 months of age),^26^ the present results indicate that children could be missing out on approximately 397 adult words (ie, 6.62 × 60 minutes), 294 vocalizations, and 68 conversational turns every day. According to the present study, as well as international estimates,^27^ children on average are exceeding these screen time guidelines. Replacing 1 hour (60 minutes) with the mean screen time children were exposed to at 36 months of age in this study (172 minutes), children could be missing out on 1139 adult words, 843 vocalizations, and 194 conversational turns per day. These estimates assume a linear association between screen time and parent-child talk; however, it may also be possible that decreases in parent-child talk occur only after a certain threshold of screen exposure is reached. This should be an important avenue of future research, to help inform updated screen time guidelines.

Having a language-rich home environment is critical to children’s language development,^2,3,4,5^ which promotes school readiness and success throughout the educational system.^28,29^ This study found a negative association between screen time and parent-child talk, which suggests that screen time is a potential mechanism that could be the basis for an intervention to promote a home environment with more parent-child talk. Implications need to be considered, however, within the reality of current family life. It is unrealistic to assume that all families will simply stop using screens with their young children. Instead, programs and policies could focus on ways to encourage families to use screen time as an opportunity for interaction with their child. The concept of interactive co-viewing has become an increasingly popular strategy for children’s screen time, demonstrating improvements in children’s language outcomes.^30^ This strategy involves parents interacting with the child during screen time to help facilitate educational benefits. When interactive co-viewing is not possible, age-appropriate, high-quality educational programs could be used that are designed in a way to facilitate the child’s language development.^30^ Encouraging these approaches within interventions aimed at promoting parent-child interactions and language exposure may alleviate some of the displacement screen time creates on opportunities for parent-child talk. Future research will aim to examine the quality of children’s screen time within the EUiLO data set to inform these approaches, which was out of the scope of the present study.

### Strengths and Limitations

This study has some strengths. It is one of few longitudinal investigations into screen time and parent-child talk in the early years of life. It is also unique in that it uses speech recognition technology to measure both screen time and parent-child talk measures. Finally, we have been able to control for a comprehensive set of confounding variables, which few studies have previously done.

Nonetheless, there are limitations that need to be acknowledged. First, this analysis did not include data from when children were 6 months of age. This omission was due to a critical confounder (parent’s self-reported psychological distress) not being measured at this wave of data collection. Given that evidence suggests parents with mental health concerns are less likely to be interacting with their young child^31^ and more likely to use screen time,^32,33^ the decision was made to not include that wave in the present analysis. There are also potential limitations within the classification of screen time based on the audio recordings extracted from the LENA software. Given that we were unable to access accompanying video footage, there is a chance that nonscreen-based electronic devices may have been miscoded as screen exposure or that some screen time was missed if there was no accompanying sound. Attempts were made to mitigate this possibility through extensive training of each researcher, and any uncertainty was checked by another researcher. Finally, some families within the study undertook their 30- or 36-month LENA recording day during the COVID-19 pandemic. Although some evidence has suggested the pandemic may have increased families’ screen time,^34^ in comparison with Australian estimates prior to the pandemic,^35^ our participants’ mean screen time does not appear to have increased substantially.

## Conclusions

Findings from this prospective cohort study suggest that increases in screen time were associated with decreases in adult words, child vocalizations, and back-and-forth interactions for children aged between 18 and 36 months, after controlling for known confounders. Interventions should focus on reducing barriers to a language-rich home environment, with a focus on supports for family’s screen time use. Identifying different ways that screen time could facilitate parent-child interactions, such as through interactive co-viewing, may be important strategies to support families given the current ubiquitous nature of screen time in families’ lives.

## Notes

Supplement.
Data Sharing Statement
