# Supplementary material for: Screen Time and Parent-Child Talk When Children Are Aged 12 to 36 Months
Source: JAMA Pediatr. 2024 Mar 4;178(4):369–75. doi: 10.1001/jamapediatrics.2023.6790 (PMC10913002; doi:10.1001/jamapediatrics.2023.6790)
Supplement: Supplement. — Data Sharing Statement [file jamapediatr-e236790-s001.pdf]

Data Sharing Statement

Brushe. Screen Time and Parent-Child Talk When Children Are Aged 12 to 36 Months. *JAMA Pediatr*. Published February 26, 2024. doi:10.1001/jamapediatrics.2023.6790

Data
Data available: No

Additional Information
Explanation for why data not available: Current ethics approvals does not permit sharing of data however, please contact the corresponding author to discuss the sharing of de-identified data.
